# Genitourinary and Syphilitic Diseases

**Published:** 1904-10

**Authors:** Nelson W. Wilson

**Affiliations:** Buffalo; Genitourinary Surgeon to the Emergency Hospital


### PROGRESS IN MEDICAL SCIENCE.

### Genitourinary and Syphilitic Diseases.

Conducted by NELSON W. WILSON, M. D., Buffalo.
Genitourinary Surgeon to the Emergency Hospital.

### CIRCUMSCRIBED FIBROSIS.

THREE cases of so-called "circumscribed fibrosis" of the cavernous bodies of the penis have come under the writer's attention recently and are now being treated. This brief résumé of these cases is in the nature of a preliminary report merely.

Very little is known concerning this rather rare condition, either of its cause or its cure, if cure there be in the uncomplicated and etiologically obscure cases. Genitourinary textbooks are either silent on the subject or dismiss it in a few words of generalisation with a hopeless prognosis. Among those who have given more than passing attention to the condition are Van Buren and Keyes, who were the first to accurately describe it; Eugene Fuller, who reports three cases; Lydston, who has had experience with four, and Taylor, Tarnowsky, Mauriac and Finger. Taylor calls the condition an "ossification" and describes it as a calcification of the sheaths of the cavernous bodies and the pectiniform septum.

In general there is no apparent cause for the condition, although all authorities agree that gout and diabetes are predisposing factors. This belief is hardly tenable, because of the comparative rarity of the condition. If gout and diabetes had much to do with its production it would certainly be more frequently met with in gouty and diabetic individuals than it is. Yet on one point every observer agrees and that is that the spongy body is never concerned in this hardening process, be it fibrosis, induration, calcification or what not.

In each of the three cases under observation there is absolutely no obtainable reason for the existence of the condition, so far as gout or diabetes are concerned. Each patient believed it due to injury during coitus or too frequent sexual indulgence. In one case there was an obscure syphilitic history; in the second, a history of rather frequent attacks of urethritis; in the third, a history absolutely free from any venereal taint. All had more or less pain on erection at a point just behind the glans on the dorsum of the penis. Examination showed indurations in each case. One was confined to the right side, the others involved both cavernous bodies, roughly heart-shaped with the apex fading off into the median line about an inch from the glans. These indurations were hard and flat with well-defined and rather sharp margins and Van Buren and Keyes' description that they "feel like cartilage" is accurate.

In the case of the patient with the history of urethritis there was reported a distinct bending forward of the glans on erection, giving the described "flail-like" appearance. The others complained only of the pain.

The syphilitic case is doing well under mercury and iodide and the induration has almost entirely disappeared. The case without history, which may be properly termed the idiopathic case, is unimproved apparently under electricity and massage with mercury vasogen inunctions; the urethritis has improved relatively 50 per cent. under iodides and massage with mercury vasogen. Of course, in connection with this general treatment, aimed directly at the condition, hygienic and sexual regulations are rigidly enforced and the urine is examined frequently.

Fuller has found the condition in men past middle life. In only one of the writer's three cases is the patient over 40. Fuller records gout and diabetes in a small percentage of cases and adds contraction of the fibers of the cavernous bodies as the third cause; but he does not pretend to give a cause for the contraction. His use of mercury has been negative of results generally speaking. Lydston calls the condition "cavernositis" and had two cases following chronic urethritis; his other cases were uncomplicated.

There have been several cases reported of diffuse induration of the cavernous bodies and the spongy body, but these were in all probability due either to urinary infiltration or thrombosis, and it is well known that cancer originating in the urethra and late syphilitic manifestation in the urethra have been mistaken for an induration of obscure origin. The rather unique case reported by Morris in 1895 wherein there was nodular hardening of all three bodies of the penis followed by priapism and death is quite likely to have been thrombotic in origin.

---

### YEAST AS AN ANTIGONORRHEIC.

ABRAHAM (*Centralblatt fur Gynakolgie,*) advances yeast as a specific in gonorrhea, but experiments made by American observers show that while yeast is deadly against all forms of cocci and kills the gonococci most rapidly it is not a specific. The many pockets and folds and the changes in the cellular elements of the urethra following gonorrhea tend to make the cure of the disease with yeast rather long drawn out and unsatisfactory. Silver still remains the best treatment in gonorrhea; argyrol, protargol, or similar preparations, in acute cases, and silver nitrate in chronic.

---

### GONORRHEA OF THE CERVIX.

Two cases of cervical gonorrhea were rapidly cured by injecting into the canal a strong solution of argyrol each day until the gonococci disappeared, as shown by microscopic examination. Then icthyol and glycerine were used for a few days, followed by a 5 per cent. solution of silver nitrate. In every case of gonorrhea in the female the cervix should be examined with great care and microscopic examination made of whatever discharge there may be.

---

### THE ABORTION OF BUBO.

SMALL (*University of Pennsylvania Medical Bulletin*) advocates the use of the following ointment for the abortion of bubo:

| | | |
|---|---|---|
| ℞ | Mercury ointment | Equal parts. |
| | Belladonna ointment | |
| | Icthyol | |
| | Lanolin | |

This should be spread on ¼ to ½ an inch thick, covered with gauze and cotton and repeated every other day. If there are not prominent signs of resolution in a reasonable length of time then hot applications may be made, which failing, an operation is necessary for the removal of the glands. The ointment is the same as suggested four or five years ago by Christian and is in practical use the only mixture which will abort a bubo. But in applying it at least three cotton bandages, three or four inches wide, must be used over the dressing and they must be put on tightly.

Burnside Foster puts his bubo cases in bed, administers a calomel purge and orders 1-12 grain of calcium sulfid every three hours with an ice bag over the bubo. Cure should be effected in a week—when it is effected, although calcium sulfid is much overrated in these cases and seldom gives the results claimed for it.

Horwitz's method of aborting bubo is to prepare the patient as for operation, make a small opening into the beginning bubo with a very small bladed bistoury and inject dioxygen and bichlorid of mercury, 1 to 5000. After the cavity is cleansed it is filled with vaseline, containing 10 per cent. of iodoform; a piece of ice is placed over the bubo until the vaseline hardens and a tight bandage is put on. In many cases where the bubo has so far advanced as to present a small area of softening, this method is almost ideal. There is no necessity for keeping the patient off his feet.

Griffith uses the Christian and Small ointment with the exception of the lanolin and uses as well ice and flaxseed poultices. He has a record of 33⅓ per cent. of abortions.

---

### GENERAL GONORRHEAL INFECTION.

Generalised gonorrhea, called fulminant by Fuller, who reports the case, is so infrequent that a brief description of his case will be interesting. In this department a few months ago a case of general infection was reported from the writer's service at the Emergency Hospital. Fuller's case, the patient, a married man, contracted gonorrhea and started West with the double object of concealing his condition from his wife and of transacting some business. He got as far as Pittsburg when he was taken ill on the train and so serious was his condition that he was removed to a hospital. His temperature ran up to 104° ; the endocardium and kidneys were affected and an arthritis developed in one shoulder. He left the hospital a wreck, with albuminous urine and a stiff shoulder.

---

### LONGEVITY OF THE GONOCOCCUS.

Cabot reports the record case of longevity of the gonococcus, provided his patient was truthful, and he sees no reason to doubt his statements. The case was that of a man who had been married fifteen years and who gave a very clear history of severe gonorrhea prior to his marriage. During his married life he had been faithful, yet five days after an intercourse with his wife a discharge appeared from the urethra which in appearance was gonorrheal. Microscopic examination showed numbers of bodies resembling gonococci. His discharge was lessened and finally cured by antigonorrheal treatment. There was no sign of infection in his wife.

Cases are recorded where the gonococcus has lived in a quiescent state for two and three years beyond any doubt, and Dr. Cabot's report is unusually interesting because of the length of time which elapsed before the inactive cocci set up a new infection and because of the change in their appearance when examined under the microscope, it being remembered that they "resembled" the gonococcus.

---

### THE TREATMENT OF EPIDIDYMITIS.

At Roosevelt Hospital epididymitis is treated as follows: With the appearance of pain or tenderness in the epididymis the patient is put to bed, given a cathartic and urinary sedatives and placed on fluid diet. A new drug, an ester of salicylic acid, is used by painting the scrotum over the affected portion; it is absorbed and appears in the circulation as sodium salicylate. The painting is done once a day and in from twelve hours to seven days there is relief from pain. The remaining swelling is reduced by the application of mercury ointment and the internal administration of iodide of potash, and the testicle is strapped.

At the Hospital for Ruptured and Crippled an application of equal parts of tincture of iodine and tincture of belladonna is used.

In personal practice the best results have been obtained from the use every day of a small amount of 10 per cent. guaiacol ointment massaged gently over the epididymis and along the cord as high as the external ring, if the tenderness extends that far. This usually relieves the pain in from 12 to 24 hours or so markedly lessens it that it is merely a discomfort rather than an agony. As soon as the acute pain has subsided the testicle is strapped with adhesive and iodide is given internally to hasten absorption.

---

### DETERMINATION OF URIC ACID IN URINE.

Dreyfus (*New York and Phila. Med. Jour.*) calls attention to the Hopkins-Folin method, which is asserted to be accurate and is now in use in several of the pathological laboratories connected with the hospitals of the Department of Public Charities of the City of New York. The following reagents are necessary:

1. A solution of 1 liter volume containing 500 grams of ammonium sulphate; 5 grams of uranium acetate; 60 c.c. of 10 per cent. acetic acid; and distilled water to bring the bulk up to 1 liter.

2. A one-twentieth normal solution of potassium permanganate.

The method is as follows: Place 300 c.c. of urine in a beaker, add 75 c.c. of ammonium sulphate reagent, and mix thoroughly. After the precipitate has settled sufficiently (say about five minutes) filter through a double folded filter. When 250 c.c. of the filtrate has passed through, this volume is divided in two portions of 125 c.c. each, to serve as a duplicate. To each portion add 5 c.c. of concentrated water of ammonia, mix thoroughly, and allow to stand over night. The precipitated ammonium urate is then transferred to a filter and washed with a 10 per cent. solution of ammonium sulphate. Then wash the precipitate with about 100 c.c. of water into the same beaker, add 15 c.c. of concentrated sulphuric acid, and immediately titrate with n-20 solution of potassium permanganate until the first permanent tinge of pink color appears. One c.c. of potassium permanganate solution equals 3.75 milligrams of uric acid. From this calculate the amount in twenty-four hours.

A shorter process, but one which is less accurate, consists in the precipitation of uric acid with ammonium chlorid (30 grams for each 100 c.c. of urine), allowing it to stand over night, then filtering off the ammonium urate, washing with cold saturated solution of ammonium chlorid, and dissolving in hot water about 100 c.c.), containing about 15 c.c. of concentrated sulphuric acid, and titrating as before.

---

### THE CAUSES OF BLACK URINE.

Garrod (*The Practitioner*) gives a very comprehensive description of black urine and its causes. Urine which is black or which may become black is excreted in cases of jaundice, especially of long standing; in hematuria, hemaglobinuria, hematoporphyrinuria, melanotic sarcoma, alkaptonuria, ochronosus, in cases where an abundance of indican is present; in phthisis only after standing a long time; in certain rare diseases of undetermined nature, and after ingestion of certain drugs and articles of diet (including carboluria).

When due to jaundice the usual tests for bile pigment will at once reveal its nature.

The blackness in hematuria is merely an exaggeration of the smoky tint and is due to the presence of much of the contained blood-pigment in the form of methemoglobin.

The term "blackwater fever" bears witness to the character of the urine, which may result from the presence of hemoglobin.

In hematoporphyrinuria the color of the urine usually resembles that of port-wine. Urine, whatever its reaction, never shows the spectrum of acid hematoporphyrin unless a mineral acid has been added to it.

True melanuria, associated with melanotic sarcoma, is a rare condition. In melanuria the urine has usually a normal color when passed but the darkening takes place from the surface downward when exposed to the air.

Ferric chlorid or nitric acid causes the cold urine to become black immediately.

In alkaptonuria the urine has the normal appearance when passed but darkens from the surface downward on standing—the addition of an alkali hastens the process. Such urine reduces Frehling's solution with the acid of heat and ammoniacal silver-nitrate solution in the cold.

In indicanuria the color is due to the higher oxidation-products of indol. Thick, cold urine blackens when $HNO_3$ is added as with true melanuria, but there is no such immediate blackening.

Black urines with tubercular diseases seem due to the indicanuria which is especially apt to occur in cases of phthisis.

Damson plums, resorcin, hydroquinin, rhubarb and senna may cause black urine. In carboluria the diagnosis can be determined by adding barium chlorid to the urine after boiling the urine with hydrochloric acid. A copious precipitate of barium sulphate is obtained. The color-reaction of phenol with ferric chlorid is not obtained in cases of carboluria.

Mention having been made of ochronosus causing black urine, the following, while not belonging to the department of genito-urinary diseases, will be interesting:

Osler, in a recent issue of the *Lancet,* remarks that blackening of the cartilages of the body was observed by Virchow in 1866, and named "ochronosus." Osler records two cases in brothers. The first was said to be diabetes in a man of 57. The condition was, however, apparently alkaptonuria of long standing, and the copper-reducing substance was not glucose. In this patient there was blackening of the sclerotics and in the cartilages of the ears. On the hands and cheeks the skin was coal black, suggesting powder marks. An interesting fact was that the patient's son had alkaptonuria. The second case, brother of the first, was also suspected of diabetes, but was also found to be suffering from alkaptonuria. He had the same blue-black color of the sclerotics and skin. Osler remarks: "These brothers presented a singularity in gait, walking with a slight bend or incline of the hips. At first I thought the elder brother had had spinal disease, but the spine was straight, and the motion of the hip-joint was perfect. He had had rheumatic pains in several joints, and there were several Heberden's nodes."

